# Supplementary material for: A direct black-hole mass measurement in a little red dot at high redshift
Source: Nature. 2026 May 27;653(8116):1017–21. doi: 10.1038/s41586-026-10579-4 (PMC13215880; doi:10.1038/s41586-026-10579-4)
Supplement: Supplementary file 1 — This file contains Supplementary Methods—additional technical verification carried out to ensure the robustness of the results and showcases fits to individual spaxels and null tests carried out on observations of a calibration star and the continuum of the source. This file also contains additional investigation into the presence of outflows in the object and technical details of the stellar-mass estimates along with electron-scattering fits to the broad line, which are secondary to the main analysis of the paper. [file 41586_2026_10579_MOESM1_ESM.pdf]

nature portfolio

https://doi.org/10.1038/s41586-026-10579-4

Supplementary information

A direct black-hole mass measurement in a little red dot at high redshift

1 Supplementary Material

1.1 Narrow line kinematics

In order to constrain the narrow line kinematics, we perform spaxel-by-spaxel fitting of the original cube to subtract the broad Hα and the continuum components. The broad Hα emission was modeled with the same shape as derived from combined spectrum in Ref. [15], consisting of two Gaussians with FWHM of 450 and 1800 km s^{-1} and containing 41% and 59% of the total broad line flux respectively. This composite profile was then scaled in the fitting procedure to reproduce the data in each spaxel. In addition to the broad line emission, the Hα profile of QSO1 also includes significant absorption. We account for this in the fitting by including an absorption profile of the form:

$$\frac{F_\lambda}{F_{\lambda;0}} = 1 - C_f + C_f e^{-\tau_\lambda}, \quad (5)$$

where $\frac{F_\lambda}{F_{\lambda;0}}$ is the ratio of transmitted to emitted fluxes, C_f is the covering factor and τ_λ - a Gaussian optical depth distribution characterized by the optical depth at the core of the line (τ_0), Doppler broadening (b) and velocity offset from the narrow emission (dv). Following the results of [15], we adopt $C_f = 0.55$, $\tau_0 = 1.9$, $b = 100$ km s^{-1} and $dv = -36$ km s^{-1} as the parameters to Equation 5. The narrow Hα line was parametrized as a Gaussian, with σ bounded between 10 and 50 km s^{-1} and peak location allowed to shift by 100 km s^{-1} relative to the redshift obtained from an aperture spectrum. As discussed further on, the single parameter broad line + absorber model does not leave structured residuals, indicating that both the broad lines and the absorber are unresolved.

After subtracting the broad line and continuum emission, we perform Bayesian fitting of the narrow Hα emission. We choose a flat prior on the FWHM of the narrow Hα line, bounded between 10 and 200 km s^{-1}, while the redshift prior was a Gaussian centered on $z = 7.0367$ [4,15] and had a FWHM of 100 km s^{-1} in velocity space. The posteriors were estimated using a Monte Carlo integrator `emcee` [59] with only spaxels for which the line was detected at $> 5\sigma$ significance considered for further analysis. The Hα emission and kinematics maps obtained as the median values of the posteriors are shown in Fig.1. The velocities were calculated from the difference between the Hα redshift in each spaxel and the median value.

We check the validity of our line fitting procedure by investigating the model-subtracted cube for position dependent residuals. No such residuals are found, which we illustrate by presenting fits to three individual representative spaxels. One of these is picked to coincide with the inferred dynamical center while the remaining two are 150 pc away (source plane) in the red and blue shifted sides, respectively.

Supplemental Materials Fig. 1 showcases the fits to the combined line profile, their residuals and fits to the BLR-subtracted narrow line profile. As can be seen there, the combined line profile fit leaves no significant residuals near the core of the line, independent of the spaxel position. As the red and blue spaxels are separated by 300 pc, the lack of significant ($> 3\sigma$) residuals around the core of the line implies that the absorption profile is unresolved as otherwise our procedure of fixing its properties would leave significant residuals on either side of the dynamical center. This is in line with the fact that the absorption is against the continuum and the BLR component of Hα emission, which are both unresolved, hence the absorption is expected to be unresolved too.

In addition to the best-fit plots, Supplemental Materials Fig. 2 and 3 showcase corner plots of the posteriors of the full line and BLR-subtracted profile fits respectively. As seen in Supplemental Materials Fig. 2, no significant degeneracies are present between the scale of the fixed BLR model and narrow Hα properties. In particular, there are no degeneracies between the "Scale" parameter (which defines the intensity of the broad component and its absorption) and the velocity of the narrow line (identified by z_{nr}, i.e. the redshift of the narrow component), which is the most critical quantity. This indicates that the two components of Hα are well decoupled by the spectral resolution of our data.

As can be seen in the figure, the narrow Hα kinematics reveal a strong velocity gradient of an amplitude of 10 km s^{-1}. This value is smaller than the spectral pixel scale of 20-25 km s^{-1}, but it is well known that with high signal-to-noise it is possible to measure offsets below the spectral and spatial resolutions [48], with an accuracy that is given by $\Delta v \propto FWHM_{LSF} \times (S/N)^{-1}$. However, wavelength calibration issues may potentially introduce spurious effects. In particular, due to data-processing issues, point sources may display spurious rotation of up to $\lesssim 1$ spectral pixel (Beck T., in prep.[⋆]). In case of false rotation, the inferred velocity gradient is always across the IFU slicers, because spaxels along the same slice tend to have a constant velocity offset with respect to the centre of the source. However, in our case, the observed velocity gradient is aligned to the slicers (green segment in Fig.1, i.e., orthogonal to the direction expected for potential, spurious rotation. We can therefore rule out the observed rotation being due to data-reduction artifacts.

While the narrow line emission is spatially extended up to 200 pc scales as found by [11], this extent is not much larger than the IFU PSF at Hα wavelengths [15]. As shown in Fig. 1, QSO1 is resolved into only two beams. We thus do not attempt to construct a rotation curve through a full line of nodes. Instead, we bin the positions of positive and negative velocity spaxels to map out the red and blue wings of the rotation curve. The bins were chosen to include approximately equal numbers of spaxels at 100 and 150 pc distances from the center of rotation (black star in Fig.1 - this was found iteratively as part of the fit, and then confirmed to be consistent with the best fit obtained by MOKA3D). We avoided binning spaxels closer than 60 pc to the center of rotation as beam-smearing dominates on scales below 120 pc. The binned velocities are obtained through an rms weighted mean of the contributing spaxels in each bin.The rotation curve in Fig.2 is obtained by simply taking the line of sight velocity, without adding in quadrature the velocity dispersion, as it is the line of sight velocity which should follow a Keplerian rotation in the case of a point mass. Hα is unresolved in the outer parts, with an upper limit on the velocity dispersion $\sigma < 10 - 20$ km s^{-1}. In the central region the apparent velocity dispersion is of the order of 25 km s^{-1}. However, the analysis performed by MOKA3D (discussed in the Methods) reveals that this central broadening is entirely due to beam smearing of the rotation curve, with an intrinsic $\sigma \sim 7$ km s^{-1} - well below the instrumental resolution. Therefore, the whole system is consistent with not being pressure-support dominated. Our velocity errors on individual spaxels are $\sim 2 - 6$ km s^{-1}, similar to what is reasonably achievable at our resolution and signal-to-noise [15].

It should be noted that using the median redshift as the velocity zero point can skew the observed rotation as the narrow Hα flux distribution is skewed towards the blue side (Fig. 1). This causes more of the pixels on the blue side to be above the S/N threshold for inclusion into the extended kinematics map, skewing the median to one side. In order to account for this bias, we force the mean velocity across both wings to be zero. Note that in the MOKA3D modelling, discussed in the Methods, the determination of the zero velocity is a

⋆ Available on jdocs.

free parameter of the global fit; the two rest frame central velocities, independently determined with the two methods, are consistent with each other.

1.2 Spectroastrometric measurements

The spatial resolution (0.1") of the NIRSpec IFU data is larger than the 30 pc scales, below which the influence on kinematics from a point mass with a few tens million masses would be most apparent. However, both spatial and spectral information present in the data can be combined to measure sizes significantly below the nominal resolution, if the signal-to-noise is high enough. The method through which this is accomplished, spectroastrometry, was first conceived to measure separations between close binary stars[49,50] and has been applied to search for BHs in the centers of galaxies by Ref. [18] and, more recently, to measure BLR sizes and BH masses in bright QSOs[61].

The spectroastrometric measurements, when carried out on an IFU cube containing an emission line involve slicing the line into several velocity channels an constructing an image of the source in each. Afterwards, centroids in each velocity channel are measured and distances to the reference zero velocity channel are computed. With sufficient signal to noise, this method can recover a rotation curve at scales smaller than the PSF by leveraging the fact that centroid accuracy for a given S/N and spatial resolution is given by $\sigma_C = 0.6\theta(S/N)^{-1}$ [48], where θ is the size of the PSF. Given the peak S/N = 12 of the narrow Hα line, our nominal centroid accuracy is thus $\sigma_C \sim 5$ mas, corresponding to 2 pc on the source plane. In practice, the measurement error is larger than this value as it is also influenced by flux errors on individual pixels making up the image.

We perform the spectroastrometric analysis using the same broad line and continuum subtracted cube as in the previous section. The centroid measurements were carried out using three velocity channels: $-40 < v < 40$ km s^{-1}, serving as the zero velocity reference point and $-100 < v < 0$ km s^{-1}, $0 < v < 100$ km s^{-1}, tracing the blue and red wings of the line respectively. The widths of these channels are equal to 2-3 spectral channels of the underlying cube and were chosen in order to maximize S/N in each channel image. The centroid positions were measured by fitting 2D quadratic polynomials (although the results are not strongly influeced by the exact centroiding routine) via `astropy`, with the errors estimated through bootstrap resampling utilizing the rms of the blank parts of the image as 1σ error on the flux. The centroid positions are shown in Extended Data Fig.1a and indicate that the centroids of the blue and red wings of the line lie on the opposite ends of the $v = 0$ channel centroid and are broadly aligned with the large scale rotation. While the exact positions of the centroids are subject to some uncertainty, the significance of the separation is 3-5σ, indicating that the measurements trace small-scale rotation. It should be noted that the $|v| = 0$ centroid in Extended Data Fig.1a does not coincide with the dynamical center inferred by `MOKA3D` modeling, this is because the overall flux distribution (Fig. 1) is asymmetric. `MOKA3D` takes into account this asymmetry (as well as any other non-radial flux distribution), however, spectroastrometry does not. As a consequence, the absolute location of the spectro-astrometry centroids is affected by this issue, but the relative position of the centroids is very precise.

The integrated narrow Hα profile is shown in Extended Data Fig. 1b and indicates some emission present at $|v| \sim 150$ km s^{-1} at about 2σ significance, however, extending the velocity bins out to 150 km s^{-1} does not impact the results.

In order to further investigate the correspondence of spectroastrometric measurements with the inferred large-scale rotation, we also split the Hα into finer velocity channels of $|v| < 30$ km s^{-1} and $30 < |v| < 60$ km s^{-1}, the smallest independent velocity bins possible at our resolution, and repeat our analysis. The considerably lower signal to noise in the finer-split channels does significantly reduce our centroiding accuracy. However, as shown in Fig.2, the extended spectroastrometric points are consistent with the measured extended velocity field and retain a strong preference for the point mass model.

Before conducting an in-depth analysis we perform a first order estimate of the virial mass of the object. The measured separation between the red and blue (±50 km s^{-1}) centroids of Hα is $24.9^{+9.4}_{-9.4}$ pc (after correcting for the shear lensing factor of 3.52). Taking half of this value as the spectroastrometric radius gives $r_{\rm spec} = 12.4^{+4.7}_{-4.7}$ pc. Combined with $v = 50$ km s^{-1} this yields $M(< r_{\rm spec}) \sin^2 i = v^2 r_{\rm spec}/G = 7.2 \times 10^6$ M$_\odot$. We repeat this estimate using the methods laid out in[18], who derive the following mass estimator:

$$M_{\rm spec} \sin^2 i = f_{\rm spec} \frac{{\rm FWHM}^2 r_{\rm spec}}{G}, \tag{6}$$

where $r_{\rm spec}$ is the spectroastrometric radius, FWHM - the observed width of the emission line analyzed and i - the inclination angle. The calibrated $f_{\rm spec}$ value is 1.0, with 0.15 dex scatter[18]. Taking the same $r_{\rm spec}$ value as above, and using FWHM $= 52^{+14}_{-14}$ km s^{-1} [15], yields $\log M_{\rm spec} \sin^2 i/{\rm M}_\odot = 6.90^{+0.23}_{-0.23}$. Consistent with the simplified estimate above and the full analysis performed later. We note that the FWHM value used in Equation 6 is only corrected for instrumental effects and hence it does include beam smearing of the rotation curve, as it should be for applying the methods given in Ref. [18]. As shown in the subsequent analysis, this beam smearing is in fact the main line broadening mechanism in QSO1, with the intrinsic line width being unresolved (see Methods).

Lastly, we perform a null test using JWST IFU observations of reference stars from PID 1537 (PI: K. Gordon). The details of this test, which shows that systematic spatial uncertainties from data calibration do not exceed 0.5 pc, are given in the following section.

1.3 Null test using calibration stars and continuum

In order to assess the robustness of our result against calibration systematics we perform a null test using JWST NIRSpec IFU observations of calibration stars. As stars are pure bright point sources, any apparent rotation shown in their data must result from calibration systematics. In order to robustly quantify such systematics, we use IFU data obtained from PID 1537 (PI: K. Gordon), reduce it with the same pipeline as our QSO1 data, with the exception of leaving the pixel scale at 0.05" as the calibration observations lack enough dithers for oversampling, and perform spectroastrometric analysis on the stellar continuum around $\lambda = 5.0\mu m$, close to where Hα of QSO1 is located. The resulting centroids are shown in Supplementary Material Fig. 4 and showcase a complete lack of appreciable spatial offset. The offsets measured between the stellar spectroastrometric points do not exceed 0.5 pc in the rest-frame of QSO1. This shows that the 10-20 pc offsets measured in our target are not due to calibration systematics and that the calibration-induced centroid uncertainties of ~ 0.5 pc are an order of magnitude lower than those arising from the PSF or signal to noise. This conclusively shows that our results are not dominated by calibration systematics.

In addition to using a calibration star, we perform a similar test on the data cube of QSO1, defining additional high velocity channels of $100 < |v| < 3000$ and $3000 < |v| < 7000$ km s^{-1}. Emission in these channels is expected to originate from the broadest part of the BLR and continuum respectively, both of which are expected to be unresolved. Spectroastrometry of these velocity channels is

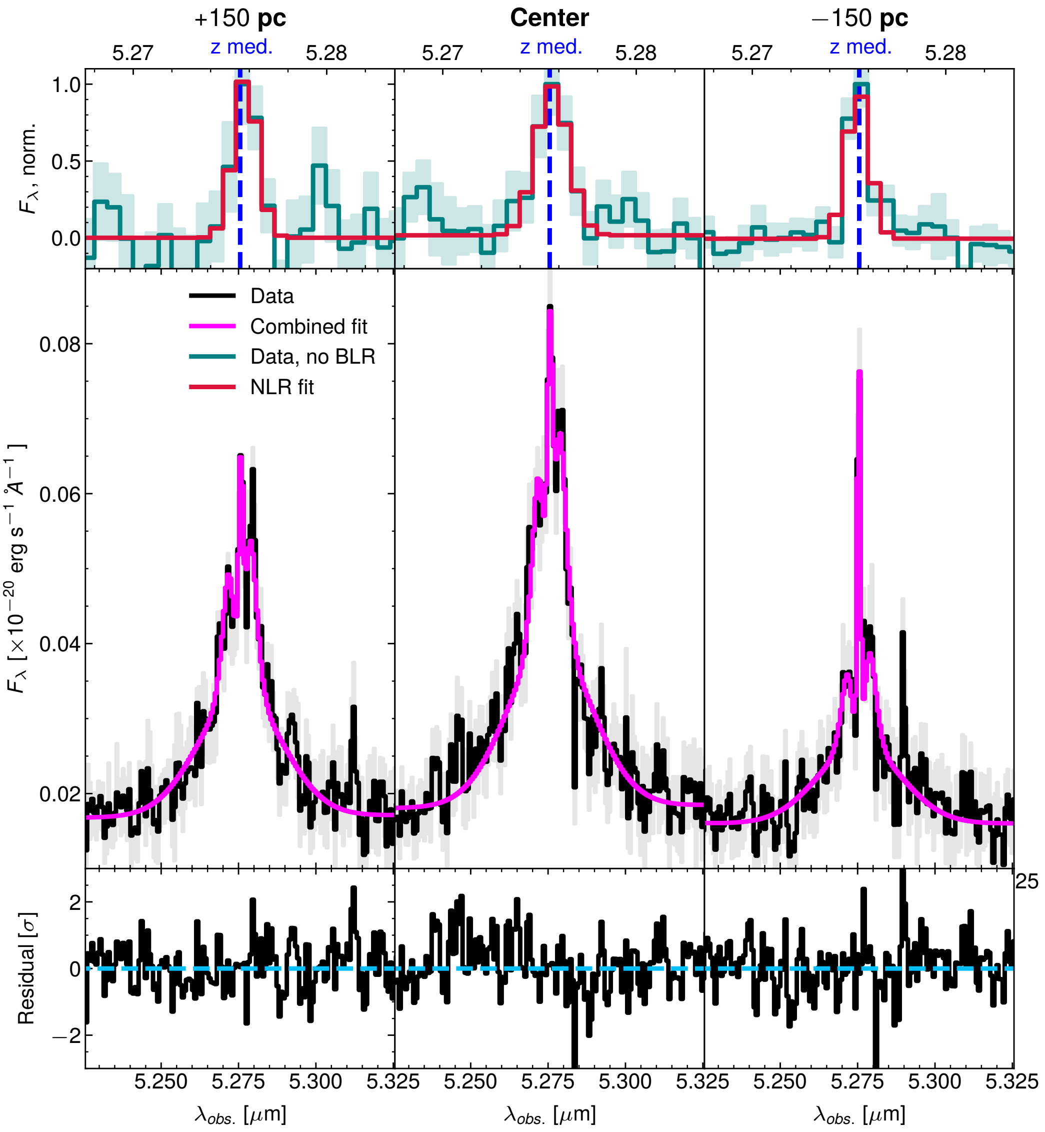

Supplementary Material Fig. 1 | Showcase of the fits to the individual spaxels. Top row: Showcase of the BLR-subtracted narrow line profiles normalized to the line peak (teal) along with the best-fit resluts (red) in the redshifted wing (+150 pc), dynamical center and blueshifted (-150 pc) wing. The dashed vertical line shows the median redshift of the data cube. The shift in the narrow line profiles clearly showcases a ~30 km s^{-1} velocity gradient across 300 pc. **Middle row:** Combined (broad and narrow) line profiles of the same spaxels (black) together with the best-fit line profiles (magenta). **Bottom row:** Residuals of the fits to the full Hα line profiles in terms of the error on each spaxel. It can be seen that our fitting procedure does not leave residuals above 2σ with the exception of a few noise spikes.

shown in Supplementary Material Fig. 5 and displays apparent offsets between the centroids of the broad lines. However, these offsets are smaller by a factor of 2-3 than those observed for the narrow line velocity channels. In addition, they lie perpendicular to the direction of the slicer, unlike the narrow line channels. This indicates that they are likely the product of inter-slicer wavelength offsets, potentially enhanced by our oversampling of the pixel scale. Regardless, this clearly showcases that the spectroastrometric signal seen in the $|v| <$ 100 km s^{-1} channels can not be ascribed to data callibration issues.

1.4 Tentative nuclear outflow

The remaining concern of our kinematics modeling is the potential presence of outflows, which are common in AGN host galaxies in the local Universe and a biconical outflow viewed edge-on can resemble a rotating disk. This is a particular concern for QSO1 as analysis of the Hβ and [OIII] emission in this source by[11] has identified a likely extended outflow component in Hβ, traced by an intermediate width, blueshifted component. In addition, the broad Hα line shows a bimodal profile, with a narrower (FWHM = 490 km s^{-1}) broad component superimposed on a broader (FWHM = 1800 km s^{-1}) emission line[15].

We investigate weather the intermediate Hα component is spatially distinct from the broad one by extracting an annular spectrum between 0.2 and 0.3" from the center - corresponding to 300 - 450 pc on the source plane. This extraction annulus is ~ 30% larger than utilized in Hβ analysis by Ref.[11] in order to account for additional beam smearing, by a 30% larger PSF at Hα wavelengths. The result-

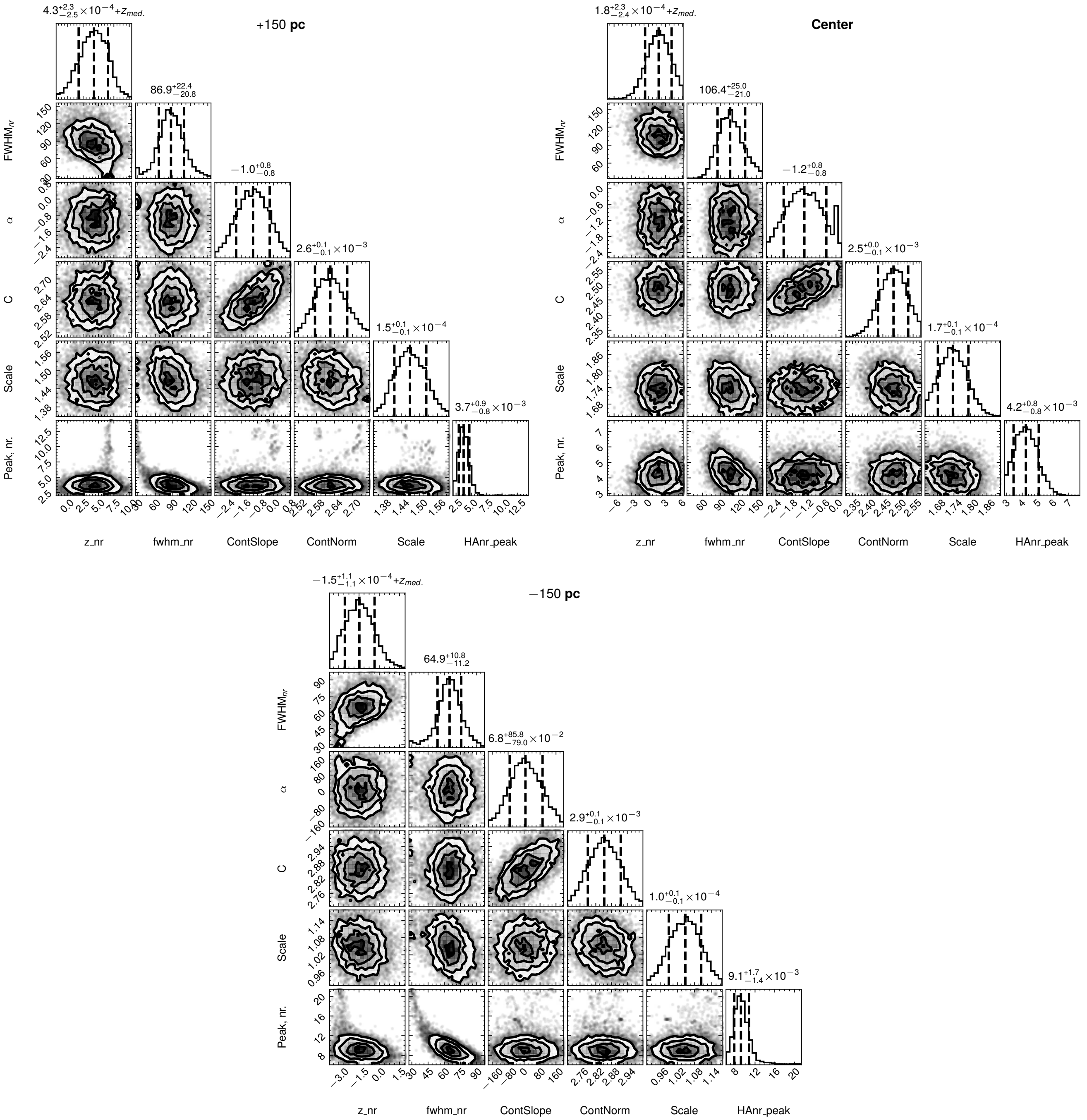

Supplementary Material Fig. 2 | Posteriors of the full line profile fits. Showcase of the corner plots of the full line profile fits fits to the same spaxels as in Supplementary Material Fig. 1. The redshift of the narrow component (z_{nr}) is given in terms of deviation from the median redshift z_{med} = 7.03601. The FWHM is given in km s^{-1}. The width of the line is given by the FWHM$_{\rm nr}$ parameter. It should be noted that for the initial model fitting we do not convolve the line width with instrumental LSF hence the values are larger than in Supplementary Material Fig. 3. The continuum power-law slope is denoted by α, while C - indicates continuum normalization. The Scale defines the scaling of the fixed-shape BLR model while Peak nr. is the amplitude of the narrow line Gaussian. No significant degeneracies are present between the scaling of the BLR model and the rest of the narrow line parameters.

ing annular spectrum is shown in Supplementary Material 6b (purple line) and clearly reveals that the intermediate velocity component (|v| < 500 km s^{-1}) is spatially distinct from the broad component and is thus likely associated with a large scale outflow.

In order to further investigate the presence of outflows in our object, we perform spectroastrometric analysis of the full line profile, splitting it into channels of $|v|$ = 50 km s^{-1}, tracing low velocity gas, $|v|$ = 250 km s^{-1} and $|v|$ = 550 km s^{-1} tracing the intermediate component and $|v|$ = 1100 km s^{-1} - tracing the high velocity broad wings. The centroids of the relevant velocity channels are shown in Supplementary Material Fig.6a and show that the red wing of the line is significantly offset from the blue. This likely indicates contamination by higher velocity unresolved outflows inducing turbulence as the red and blue nodes of the high velocity channels do not lie on

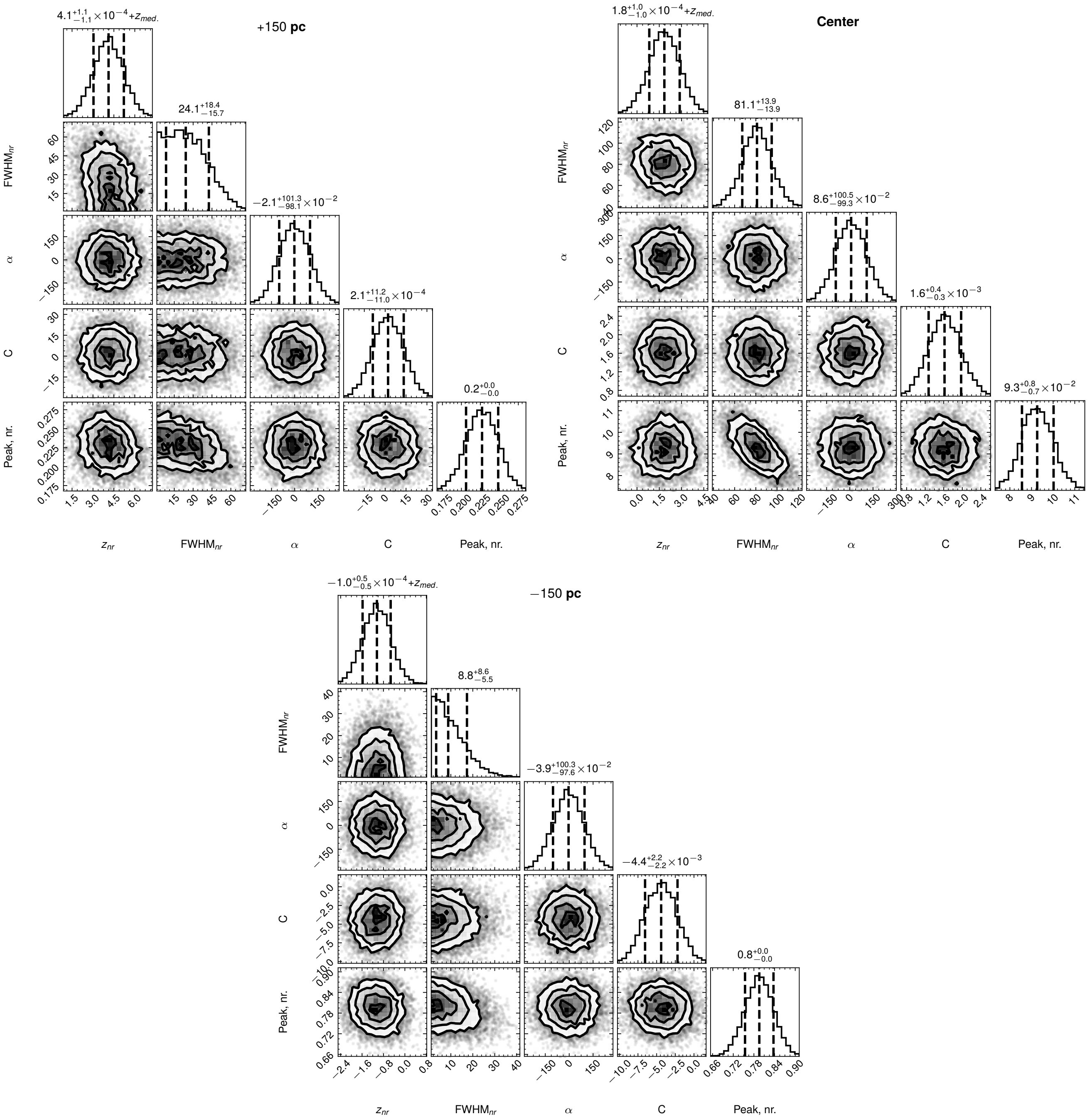

Supplementary Material Fig. 3 | Posteriors of the fits to the BLR-subtracted Hα **line.** Showcase of the corner plots of the fits to BLR-subtracted spectra of the selected spaxels. The notation is the same as in Supplementary Material Fig. 2, except absent is the 'Scale' variable, which controlled the normalization of the fixed BLR model in the combined line profile fits. The measured FWHM of the narrow Hα shown in this figure is the intrinsic value, measured accounting for the LSF. Thus, the posteriors clearly show that Hα becomes entirely unresolved in the wings while remaining clearly resolved near the dynamical center.

opposite sides of the $|v| = 0$ km s^{-1} channel. In addition, the apparent 'rotation' is more perpendicular to the IFU slices, which might be indicative of instrumental effects associated with the calibration of different slices.

While all broad components are removed by our broad line subtraction procedure, the narrow line kinematics may still end up contaminated. Indeed, the centroid of the red wing narrow line slightly shifts in position once the underlying broad emission is subtracted (see Extended Data Fig. 1), although the significance of this shift is $< 2\sigma$. However, as shown in Fig. 1, the observed velocity dispersion of the narrow component is very low and drops off with increasing distance from the axis of rotation, which would be unusual for an extended outflow. In addition, it would be a very improbable conspiracy for the velocity distribution of the putative outflow to exactly mimic Keplerian rotation. In the following section we present an equivalent MOKA3D analysis, showing that the kinematics maps are entirely inconsistent with an outflow.

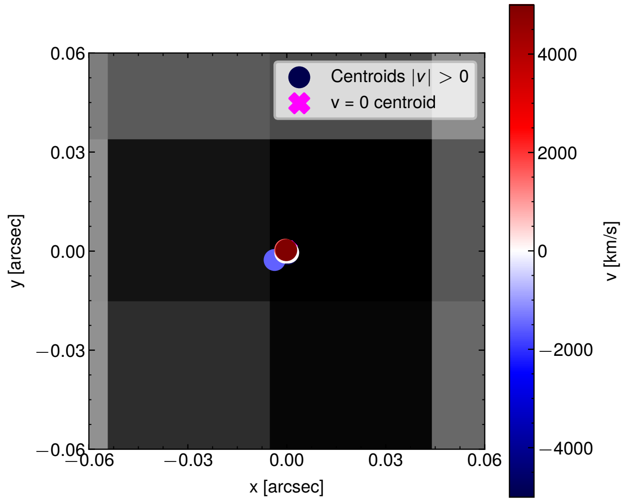

Supplementary Material Fig. 4 | Spectroastrometry of a calibration star. Centroids obtained for a calibration star showing that the values are all less than 0.1 px away from $v = 0$.

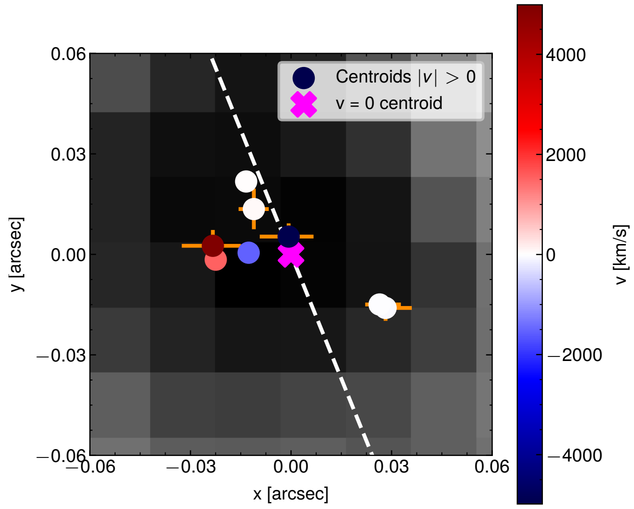

Supplementary Material Fig. 5 | Spectroastrometry of the continuum of QSO1. Extended spectroastrometric analysis of QSO1, incorporating high velocity channels tracing the edges of the BLR profile and pure continuum. The white dashed line indicates the position angle of the IFU slicer. It can be seen that the continuum and pure BLR channels have a much smaller offset than those tracing the narrow line and that their offsets are perpendicular to the slicer, unlike what is seen in narrow line emission.

1.5 Ruling out an outflow scenario for the narrow line extended kinematics

Here we investigate more quantitatively the possibility that the narrow line kinematics is due to a large scale outflow. We construct a biconical outflow model in MOKA3D. In particular, we assume a bi-conical

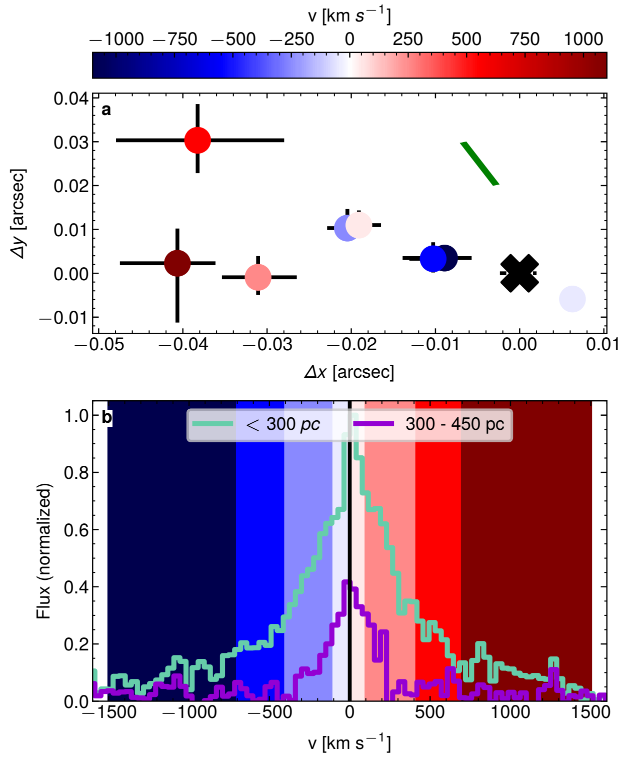

Supplementary Material Fig. 6 | Spectroastrometric analysis of the full line profile. Panel **a** Centroids of the different velocity channels defined in the text showcasing the offset of the positive high velocity channels. The $|v| = 0$ channel is marked with a black cross while the green line shows the PA of the IFU slicer. Panel **b** Hα line profiles extracted from the inner 300 pc (green line) and an 300 - 450 pc annulus (violet line) normalized to the peak of the inner 300 pc spectrum. The vertical colored bars denote the velocity bins for which centroids were measured with colors corresponding to their points in **a**.

geometry with aperture angle of 40°, found iteratively through testing different values until one matching the data was found, position angle of 45°[†] (although we note that leaving the position angle free does not change the results) and a constant radial velocity field for both the approaching and receding cones. The MOKA3D free parameters in the following analysis are two: the inclination with respect to the line of sight and the intrinsic radial outflow velocity (see Extended Data Tab. 1). We constrained the inclination angle of the blue-shifted cone to be directed towards the observer. In particular, we allowed the inclination to vary within the range [0°,90°], with the two boundaries representing a cone with the axis directed along the line of sight or lying on the plane on the sky, respectively. Based on the observed projected velocity range in moment maps in Fig.1 and the possible inclination range, we allowed the intrinsic outflow radial velocity to vary within a conservative range of [0, 200] km s^{-1}. As a result, the best-fit parameters are an outflow intrinsic velocity of 110 km s^{-1} and an inclination with respect to the line of sight of 85°, i.e. only 5° inclination with respect to the plane of the sky, which is the expected geometrical configuration that would mimic a rotating disc. Then, we constructed the best-fit model cube adopting the mentioned parameters and compared the resulting 2D moment maps with the observed

[†] The position angle is measured clockwise from the north.

ones in Fig. 1. As shown in Supplementary Material Fig. 7, the bi-conical outflow model still leaves systematic residuals resembling a rotating disk and results in $\chi^2_R = 9.6$, which is considerably worse than any of the models assuming a rotating disk. We notice that the flux map in Supplementary Material Fig. 7 shows larger residuals close to the cone apex. This has to be ascribed to conical geometry of the model which, by construction, cannot reproduce the observed features that are likely associated to purely circular motions, not considered in the current purely radial outflow model. Similarly, the LOS velocity map shows systematic residuals resembling a rotating disc which directly indicates the impossibility of reproducing the observed features with a purely radial outflow. Therefore, if potential outflow contamination to the narrow line kinematics is present, it cannot be reproduced by a simple bi-conical outflow with a purely radial velocity profile. In order to reproduce the observed velocity profile one would have to incorporate an outflow kinematical model that would mimic a rotation pattern.

We also note that the velocities observed in the large scale emission (about 10 km s^{-1}) are much lower than what seen in outflows driven by AGN with luminosities ($10^{44} - 10^{45}$ $erg\ s^{-1}$) similar to QSO1, which are in excess of about 400 km s^{-1} (e.g. Refs. [62,63]).

1.6 BH mass estimate assuming electron scattering

In order to test how the BH mass would change if the dominant broadening mechanism was electron scattering, as proposed by Ref. [7], we refit the Hα and Hβ lines of QSO1 with a scattering profile constructed via the following equation:

$$P(\lambda) = f_{\rm scatt}(E * G_{\rm BLR})(\lambda) + (1 - f_{\rm scatt})G_{\rm BLR}(\lambda), \tag{7}$$

where $G_{\rm BLR}(\lambda)$ is the intrinsic broad-line profile, assumed to be virially broadened and Gaussian in shape, $f_{\rm scatt}$ - the fraction of light affected by the electron scattering and $E(\lambda)$ - the exponential electron scattering profile [64], defined as

$$E(\lambda) \propto e^{-\frac{|\lambda-\lambda_0|}{W}}, \tag{8}$$

where λ_0 is the central wavelength, and W is the width of the exponential.

The absorption features in the Balmer lines were modeled using Equation 1, as in the previous section. The priors on the fit were uninformative, flat priors over all model parameters. We inspected the posterior probability over each model parameter, and verified that no posterior hit the boundaries (except for physically motivated boundaries, such as non-negative emission-line flux). The posteriors were estimated using the Monte Carlo sampler `emcee` [59], using ten walkers per free parameters, and initializing the chains inside a sphere centered on the maximum-likelihood solution, obtained from differential evolution [65]. The best-fit, produced by the medians of the posteriors, is shown in Supplementary material Fig. 8. As can be seen there, the integrated spectrum is well fitted by this model producing a BH mass estimate of $\log M_{\rm BH}/M_\odot = 5.9 \pm 0.1$. We note that, as discussed in the main text and shown in Fig. 3, this value is nearly 2 dex lower than the direct mass estimates, showing that it is implausible that the observed Hα line profile can be attributed to electron scattering. In case of the BH mass being this low, extreme stellar densities would be required to produce a point mass consistent with the one measured. This result confirms the findings of Ref. [66]: despite providing an excellent fit to the data, the electron scattering model violates basic physical constraints.

1.7 Dynamic upper limit on the stellar mass in the host galaxy

In addition to constraining the BH mass, we endeavor to estimate the maximum extended mass component permitted by our data. For a conservative upper limit, for this analysis we add the velocity dispersion (σ) in quadrature to the velocity of each spaxel and redo the binning procedure. This curve represents the maximal rotation allowed in the extended Hα region and thus provides the highest upper limit on any extended mass component. We perform this estimate by constructing a combined point mass and an extended exponential disc component modelled as an $n = 1$ Sérsic density profile of half mass radius 100 pc, truncated at 300 pc - the maximum extent of the source found by Ref. [11]. We note that galaxies with such a small stellar mass at such high redshift are observed to be on average smaller than this radius [60].

If both components are left free, the point mass dominates, essentially recovering the previous pure Keplerian fits. We thus derive an upper limit on the extended mass component by forcing a particular extended mass value and increasing it until the $\Delta BIC > 5$ between the fiducial and extended component models. Following this method, we derive the maximum extended mass of $\log M \sin^2 i = 6.85$, similar to the mass of the BH, which is increased to $\log M \sin^2 i = 6.94$ by the higher velocity field.

However, this extended mass component is a composite of contributions from stars, gas and Dark Matter and thus only serves as an upper limit to the total stellar mass. We tighten this limit further by estimating the ionized gas mass from the narrow line emission. The ionized gas mass can be estimated from narrow Hβ emission via the following equation:

$$M_{\rm ion} = 0.8\frac{m_{\rm p}\, L_{\rm H\beta}}{j_{\rm H\beta}\, n_{\rm e}}, \tag{9}$$

where $L_{\rm H\beta}$ is the Hβ line luminosity, $j_{\rm H\beta}$ - Hβ emissivity, n_e - electron density and m_p - proton mass. Assuming standard warm interstellar medium conditions of $T \sim 10000$ K and $n_e \sim 100\,{\rm cm}^{-3}$ and taking $L_{H\beta} = 5 \times 10^{40}$ erg s^{-1} from [4] we obtain $\log M_{ion}/M_\odot = 6.7$, with a similar mass obtained by using narrow Hα measurements from [15]. Utilizing the inclination of $52 \pm 2°$ derived from `MOKA3D`, we estimate that the effective contribution of ionized gas to the uncorrected kinematics is $\log M_{ion} \sin^2 i = 6.5$. Hence, the dynamical contribution of an extended stellar component cannot exceed $\log M \sin^2 i - M_{ion} \sin^2 i = 6.6$.

In addition to an extended stellar distribution we also consider the maximal contribution of a compact NSC. For this, we fix R_c to 0.5 pc, a factor of 2 below the most compact NSCs seen in the local Universe [51] to account for higher redshift structures being less relaxed. As with the previous attempts to fit a non-point mass distribution, the mass of the extended component goes to zero if left free. We thus estimate an upper limit on the NSC mass through requiring $\Delta BIC < 5$ from the fiducial model. The upper limit on the NSC mass is thus $M(< R_c) \sin^2 i = 10^{6.1}$ $M_\odot$ with the BH mass reducing slightly to $M_{\rm BH} \sin^2 i = 10^{6.44}$ $M_\odot$. A similar mass ratio is maintained even when setting $R_c = 0.2$ pc, the upper limit found earlier.

We also repeat the above analysis using the `MOKA3D` framework, finding that $M_* \leq 10^{7.2}$ $M_\odot$ within ~800 pc (i.e. well beyond any possible galaxy extension at this redshift) [60] for an extended exponential disc profile and $M_* \leq 10^{6.55}$ $M_\odot$ for a NSC-like distribution with $R_c = 0.5$ pc. Taking the estimated $M_{\rm BH} = 10^{7.7}$ $M_\odot$, we obtain $M_{BH}/M_{star} > 2 - 15$. As before, the stellar masses estimated above are upper limits, as dynamical mass measures are sensitive to the combined mass of gas, stars and DM, which we are unable to fully disentangle due to lack of emission tracing gas on larger scales (but

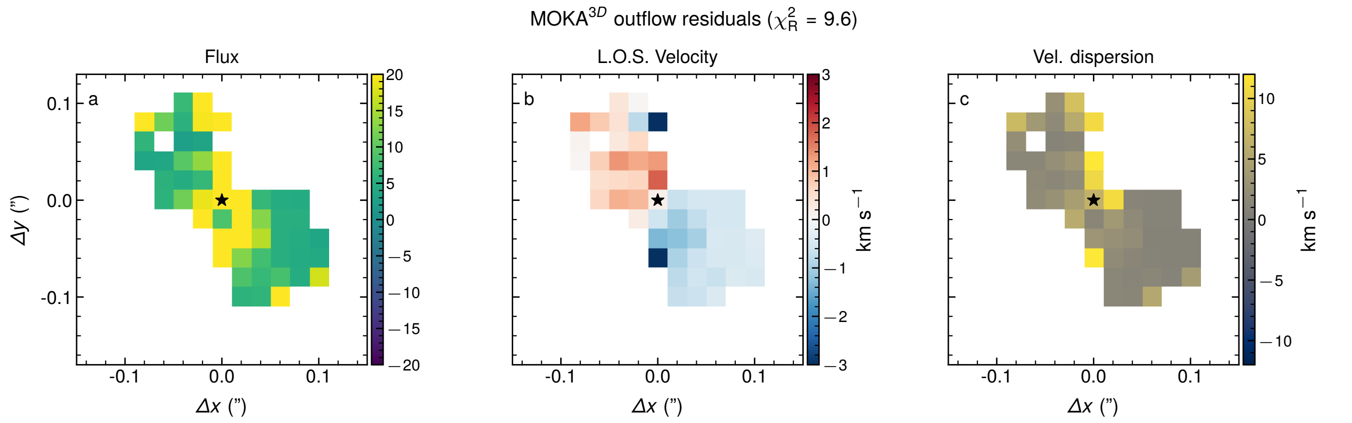

Supplementary Material Fig. 7 | Residuals of a fit assuming a biconical outflow. The residuals and hence the χ_R^2 value are considerably worse than any of the fits assuming a rotating disk.

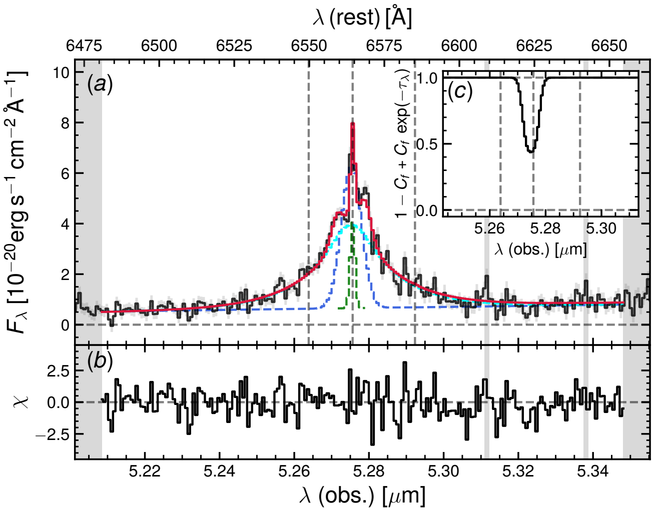

Supplementary Material Fig. 8 | Fits to the Hα **line assuming a line profile dominated by electron scattering.** The combined best fit is shown by the red line, the scattered Hα BLR is shown in cyan with the NLR shown in green, the blue line shows the transmitted, unscattered BLR. As can be seen in residual plots (panel b), the model fits the data well, however, the BH mass derived is 2 dex ($\sim 4\sigma$) below the dynamic mass measurements as discussed in the text.

see the following section for additional spectroscopic constraints on the stellar and gas masses). We note that any extended mass component below 10^6 $M_\odot$ has no impact on the fit given the observational uncertainties.

The above analysis implies a $M_{\rm BH}/M_{star} > 2$ for any reasonable mass distribution, with the inclination term canceling in the ratio. This is an extreme lower limit since it does not include contributions from non-ionized gas and DM. Therefore, QSO1 represents the most naked BH yet discovered, leaving only direct collapse and primordial BHs as potential progenitors. These findings corroborate our initial back of the envelope estimate of the BH sphere of influence for which, in the absence of stellar features, we use the σ_N of the narrow lines as a proxy for σ_* [67] and find that the entirety of the object lies within this sphere.

Aside from heavy seeding, a lone black hole can originate as a runaway from a triple black hole interaction in a galaxy nucleus [68,69]. The lightest black hole is ejected at about 1000 km s^{-1}, and so at redshift 7 may travel up to 20 arcsec in 100 Myr. We consider this scenario very unlikely as it compounds 2 unlikely events (being ejected and then coinciding with a cluster caustic). Searching the neighborhood of QSO1 for a suitable galaxy is complicated by the strong lensing. However, Ref. [12] do not report any nearby objects associated with QSO1. Either way, a quantitative examination into the origins of this object is beyond the scope of this paper.

1.8 Spectroscopic constraints on the gas and stellar masses

The dynamical mass estimates discussed above are sensitive to the total mass present in the object. The kinematics fits shown in Fig.1-2 alone constitute $> 5\sigma$ evidence against significant stellar contribution to the mass of the object. In addition, Ref. [4] have shown that the optical continuum of QSO1 is dominated by AGN emission, with a stellar contribution of <10%. Stellar contribution to the UV continuum can not be ruled out and was indeed suggested as a way to explain the observed power law slopes by Ref. [8], and the observed spatial offsets [70], although some indications of UV variability in QSO1 [4] suggest that even the UV maybe AGN-dominated. Yet, even conservatively assuming that the UV light is entirely stellar, it would give a stellar mass much lower than the dynamical mass inferred earlier, as discussed in the following. The UV magnitude measured for image A is $M_{\rm UV} = -15.60$, after correction of $\mu = 6.2$. Utilizing the $M_* - M_{\rm UV}$ relation from Ref. [71], we obtain a stellar mass estimate of $\log M_*/{\rm M}_\odot = 6.03$. This is almost 1 dex lower than the measured virial mass. In addition, while Ref. [4] have shown that the UV continuum is damped by a different hydrogen column density than the optical, the observed slope can be explained by nebular emission. Hence our stellar mass value should be treated as an upper limit and stars can be conclusively ruled out as dominant contributors to the virial mass budget.

As discussed in the previous section, contributions to the dynamical mass from ionized ISM gas, on scales of $\sim$ 100 pc, are very small, and anyhow are taken into account. Contribution, on these scales from neutral gas, would make the upper limit on the stellar mass (based on dynamical modeling) even lower.

In the nuclear region, the amount of ionized gas is even smaller.

Indeed, as the emissivity scales quadratically with the density and the broad lines are coming from the BLR, which has a density higher than 10^9 cm^{-3}, the mass of nuclear ionized gas inferred from the luminosity of the broad Balmer lines is only of order of a few solar masses (see e.g. Ref. [72]), as also inferred from Equation 9.

The putative envelope of dense ionized gas that, according to the electron scattering scenarios, should produce the exponential line wings must also have a negligible mass. Indeed, such gas must recombine and its signature should be associated with one of the Balmer emission line components and, if the densities are as high as expected by those scenarios, the masses must be similarly low. Ref[7] estimate an upper limit on the mass of the putative ionized envelope of $< 10^5$ $M_\odot$ (for systems that are much more luminous than QSO1), i.e. negligible relative to the mass estimated by us dynamically.

The envelope of neutral gas responsible for the putative Balmer scattering[8,73] must also be negligible. This can be estimated by scaling from the case of the Rosetta Stone[5], in which the column and density of neutral gas was estimated in detail, thanks to the presence of multiple transitions in absorption. In this case we obtain a mass of $\sim 6 \times 10^4$ $M_\odot$, again negligible. In the case of the local LRD analogue the "Lord of LRDs"[74] or the "Egg"[75], which has a luminosity similar to QSO1, an upper limit on the mass of the neutral envelope of 7×10^5 $M_\odot$ has been estimated - again negligible when compared to our dynamical mass estimates.

References (continued)

61. Abuter R., et al., 2024. A dynamical measure of the black hole mass in a quasar 11 billion years ago, Nature, 627, 281
62. Fiore F., et al., 2017. AGN wind scaling relations and the co-evolution of black holes and galaxies, A&A, 601, A143
63. Fluetsch A., et al., 2021. Properties of the multiphase outflows in local (ultra)luminous infrared galaxies, MNRAS, 505, 5753
64. Laor A., 2006. Evidence for Line Broadening by Electron Scattering in the Broad-Line Region of NGC 4395, ApJ, 643, 112
65. Storn R., Price K., 1997. Differential evolution–a simple and efficient heuristic for global optimization over continuous spaces, Journal of global optimization, 11, 341
66. Brazzini M., et al., 2025. Ruling out dominant electron scattering in Little Red Dots' Rosetta Stone using multiple hydrogen lines, p. arXiv:2507.08929 arXiv:2507.08929
67. Bezanson R., et al., 2018. 1D Kinematics from Stars and Ionized Gas at z ∼ 0.8 from the LEGA-C Spectroscopic Survey of Massive Galaxies, ApJ, 868, L36
68. Saslaw W. C., Valtonen M. J., Aarseth S. J., 1974. The Gravitational Slingshot and the Structure of Extragalactic Radio Sources, ApJ, 190, 253
69. Volonteri M., Madau P., Haardt F., 2003. The Formation of Galaxy Stellar Cores by the Hierarchical Merging of Supermassive Black Holes, ApJ, 593, 661
70. Torralba A., et al., 2025. A weak Lyα halo for an extremely bright Little Red Dot. Indications of enshrouded SMBH growth, p. arXiv:2505.09542 arXiv:2505.09542
71. Simmonds C., et al., 2024. Ionizing properties of galaxies in JADES for a stellar mass complete sample: resolving the cosmic ionizing photon budget crisis at the Epoch of Reionization, MNRAS, 535, 2998
72. Maiolino R., et al., 2024. A small and vigorous black hole in the early Universe, Nature, 627, 59
73. Chang S.-J., Gronke M., Matthee J., Mason C., 2025. Impact of Resonance, Raman, and Thomson Scattering on Hydrogen Line Formation in Little Red Dots, p. arXiv:2508.08768 arXiv:2508.08768
74. Ji X., et al., 2025. Lord of LRDs: Insights into a "Little Red Dot" with a low-ionization spectrum at z = 0.1, p. arXiv:2507.23774 arXiv:2507.23774
75. Lin X., et al., 2025. The Discovery of Little Red Dots in the Local Universe: Signatures of Cool Gas Envelopes, p. arXiv:2507.10659 arXiv:2507.10659
